# *In vivo* detection of murine glioblastoma through Raman and reflectance fiber-probe spectroscopies

**DOI:** 10.1117/1.NPh.7.4.045010

**Published:** 2020-12-01

**Authors:** Enrico Baria, Enrico Pracucci, Vinoshene Pillai, Francesco S. Pavone, Gian M. Ratto, Riccardo Cicchi

**Affiliations:** a University of Florence, Department of Physics, Sesto Fiorentino, Italy; b European Laboratory for Non-Linear Spectroscopy, Sesto Fiorentino, Italy; c Scuola Normale Superiore, National Enterprise for Nanoscience and Nanotechnology, Pisa, Italy; d National Institute of Optics – National Research Council, Sesto Fiorentino, Italy

**Keywords:** glioblastoma, spectroscopy, *in-vivo*, Raman, fluorescence, reflectance

## Abstract

**Significance:** Glioblastoma (GBM) is the most common and aggressive malignant brain tumor in adults. With a worldwide incidence rate of 2 to 3 per 100,000 people, it accounts for more than 60% of all brain cancers; currently, its 5-year survival rate is <5%. GBM treatment relies mainly on surgical resection. In this framework, multimodal optical spectroscopy could provide a fast and label-free tool for improving tumor detection and guiding the removal of diseased tissues.

**Aim:** Discriminating healthy brain from GBM tissues in an animal model through the combination of Raman and reflectance spectroscopies.

**Approach:** EGFP-GL261 cells were injected into the brains of eight laboratory mice for inducing murine GBM in these animals. A multimodal optical fiber probe combining fluorescence, Raman, and reflectance spectroscopy was used to localize *in vivo* healthy and tumor brain areas and to collect their spectral information.

**Results:** Tumor areas were localized through the detection of EGFP fluorescence emission. Then, Raman and reflectance spectra were collected from healthy and tumor tissues, and later analyzed through principal component analysis and linear discriminant analysis in order to develop a classification algorithm. Raman and reflectance spectra resulted in 92% and 93% classification accuracy, respectively. Combining together these techniques allowed improving the discrimination between healthy and tumor tissues up to 97%.

**Conclusions:** These preliminary results demonstrate the potential of multimodal fiber-probe spectroscopy for *in vivo* label-free detection and delineation of brain tumors, and thus represent an additional, encouraging step toward clinical translation and deployment of fiber-probe spectroscopy.

## 1 Introduction

### 1.1 Glioblastoma

Glioblastoma (GBM) is the most common and aggressive malignant brain tumor in adults, accounting for more than 60% of all cases.^1^ Current treatment options at diagnosis include surgical resection, radiation, and chemotherapy^2^ but—despite advances in this field—it remains largely incurable; its 5-year survival rate^3^ is <5%. Extensive and complete surgical resection of GBM is difficult due to the high invasiveness of these tumors. However, multiple studies^4^^,^^5^ have demonstrated the importance of aggressive surgical resection—when possible—with trends toward better outcomes in those patients with a greater extent of resection.

### 1.2 Multimodal Optical Spectroscopy

In this framework, multimodal optical spectroscopy could provide a fast and label-free tool for improving tumor detection and guiding the removal of diseased tissues.^6^^–^^8^ In fact, laser-induced fluorescence^9^ spectroscopy of tissue native autofluorescent molecules has been widely used to discriminate normal and diseased tissues. This method has the ability to provide quantitative and real-time biochemical information related to tissue pathology and has the potential for minimally invasive diagnosis, considering the recent advancements in fiber-optic probes. Moreover, diffuse reflectance and Raman spectroscopy could provide additional information for improving the diagnosis of tumors. Diffuse reflectance^10^ employs a broadband light source and it is based on the ratiometric measurement of the spectrum of light back-scattered by the tissue, as compared with the illumination spectrum. The measured spectrum allows characterizing tissue chromophores and scatterers,^11^ which in turn are related to the physiologic conditions of the tissue under investigation. On the other hand, Raman spectroscopy^12^ is based on inelastic scattering processes in which non-resonant photons contribute to the excitation of vibrational molecular modes. The measurement of the energy lost in the process allows measuring a vibrational spectrum, typical of the specific molecule involved in the process.

Hence, the multimodal combination of different spectroscopic techniques^13^^,^^14^ allows investigating tissue composition, either at structural, molecular, and metabolic level, without recurring to labels and in a relatively small amount of time (˜seconds, or even less). Moreover, machine learning^15^^,^^16^ methods can be used for providing unbiased, operator-independent, and automated clinical/biomedical classification based on the spectral data collected with such approach.

### 1.3 *In Vivo* Detection of GBM

In this proof-of-concept study, we used multimodal fiber-probe spectroscopy for *in vivo* examination of mouse brain tumors induced by murine GBM cell line. In particular, we used label-guided fluorescence for detecting tumor areas and, then, Raman and reflectance spectroscopies for examining and classifying both healthy and diseased tissues. Finally, we developed a scoring algorithm based on principal component analysis (PCA) and linear discriminant analysis (LDA) for discriminating the two tissue types based on their spectral profiles.

## 2 Materials and Methods

The main goal of this study was to explore the use of Raman and reflectance fiber-probe spectroscopies for *in vivo* detection of GBM in an animal model (i.e., laboratory mice). The following sections will describe animal preparation, our experimental setups, data collection, and analysis.

### 2.1 Animal Model

We induced the development of a glioma tumor in laboratory mice in order to compare the spectra of healthy and diseased brain tissues, as described in the following paragraphs. All animal care and experimental procedures were performed in strict accordance with the recommendations of the Italian Ministry of Health (Dlg. 26/14) and according to protocol 143-2017 PR approved by the Ministry of Health on February 13, 2017.

#### 2.1.1 Cell culture preparation

The experiment was conducted using EGFP-GL261 tumor cells, a murine glioma cell line with characteristics similar to human GBM and with stable expression of the green fluorescent protein (EGFP). GL261 cells were transfected with EGFP using the electroporation technique. The cells were detached using 0.05% trypsin-EDTA (Gibco), washed with 5 ml of PBS and centrifuged at 1200 rpm for 5 min; then, they were re-suspended in 120  μl of resuspension buffer (Invitrogen) and 5  μl of DNA were added. Afterward, the cells were electroporated with two 20-ms pulses at 1200 V using Neon Transfection System 100  μl pipette tips (ThermoFisher Scientific). The amount of DNA used is around 10 to 20  μg for each electroporation. The transfected cells were plated and maintained in Dulbecco’s modified Eagle’s medium (DMEM, Gibco) containing 10% fetal bovine serum, 1% penicillin–streptomycin (100  units/ml of penicillin and 100  μg/ml of streptomycin, Gibco), 1 mM sodium pyruvate (Gibco) and 10 mM HEPES (Gibco) in a humidified atmosphere of 5% ${\mathrm{CO}}_{2}$. The culture medium was replaced with fresh medium 2 to 3 times per week. Once the confluence reaches to about 90%, the cells were harvested using 0.05% trypsin-EDTA. The cells were pelleted by centrifugation at 1200 rpm for 5 min (Sorvall T3 Centrifuge, ThermoScientfic). The pellet was re-suspended with PBS and the resulting solution was injected into the mouse brain using a glass capillary.

#### 2.1.2 Mice preparation

Adult C57BL/6 mice were maintained at a mice facility with controlled temperature and light, where they received food and water *ad libitum*. The animals were anesthetized with Avertin (0.2  ml/g body weight) by intraperitoneal injection. Hair at the top of the head was removed using a hair depilatory cream and fixed on the stereotaxic stage. A single skin cut of ∼0.5 to 1 cm was made using a pair of scissors and the exposed sub-cutaneous tissue was gently removed. A hand drill was used to thin the skull at the injection site, which is the visual cortex. Two microliters of EGFP-GL261 cell suspension containing ∼40,000  cells in total were aspired into the glass capillary and slowly injected into the mouse cortex. The glass capillary was inserted at 300  μm at first and then retracted ∼50  μm before injection of cell suspension. A total of 5 min was spent for the injection and additional 2 min before removing the glass capillary. The surface of the injection site was then washed with PBS and skin was closed with simple stitches. Iodine antiseptic cream was applied on the stitches to prevent infection. To relieve pain, 5 drops of paracetamol (100  mg/ml) per 100 ml of drinking water was provided for the animals after surgery. All the instruments used were sterilized.

Another surgery was carried out under anesthesia induced by Avertin (0.2  ml/g body weight) for placing a cranial window. The mice were placed on a heating pad and fixed on the stereotaxic stage. A longitudinal incision of the skin was performed between the occiput and the forehead, followed by skin and sub-cutaneous tissue removal around the injected site. Using a hand drill, the skull was thinned to open a 5-mm-diameter window. Forceps were used to break and remove the skull gently. The dura mater was continuously rinsed with artificial cerebro-spinal fluid: 125 mM NaCl, 25 mM ${\mathrm{NaHCO}}_{3}$, 1 mM ${\mathrm{MgCl}}_{2}$, 2.5 mM KCl, 1.25 ${\mathrm{KH}}_{2}{\mathrm{PO}}_{4}$, 2 mM ${\mathrm{CaCl}}_{2}*H_{2}O$, and 10 mM glucose. A coverslip (5-mm diameter and 0.17-mm thickness) was used to cover the window and fixed using strong adhesive glue and self-curing dental cement carefully. We chose a quartz coverslip because, when excited at 785 nm, standard glass emits fluorescence at wavelengths corresponding to the “fingerprint region” of Raman spectroscopy. Again, all the instruments used were sterilized.

Finally, another cranial window was placed at a certain distance from the injection site, in order to have an optical access to healthy brain tissue.

#### 2.1.3 Optical measurements

**Fig. 1:**
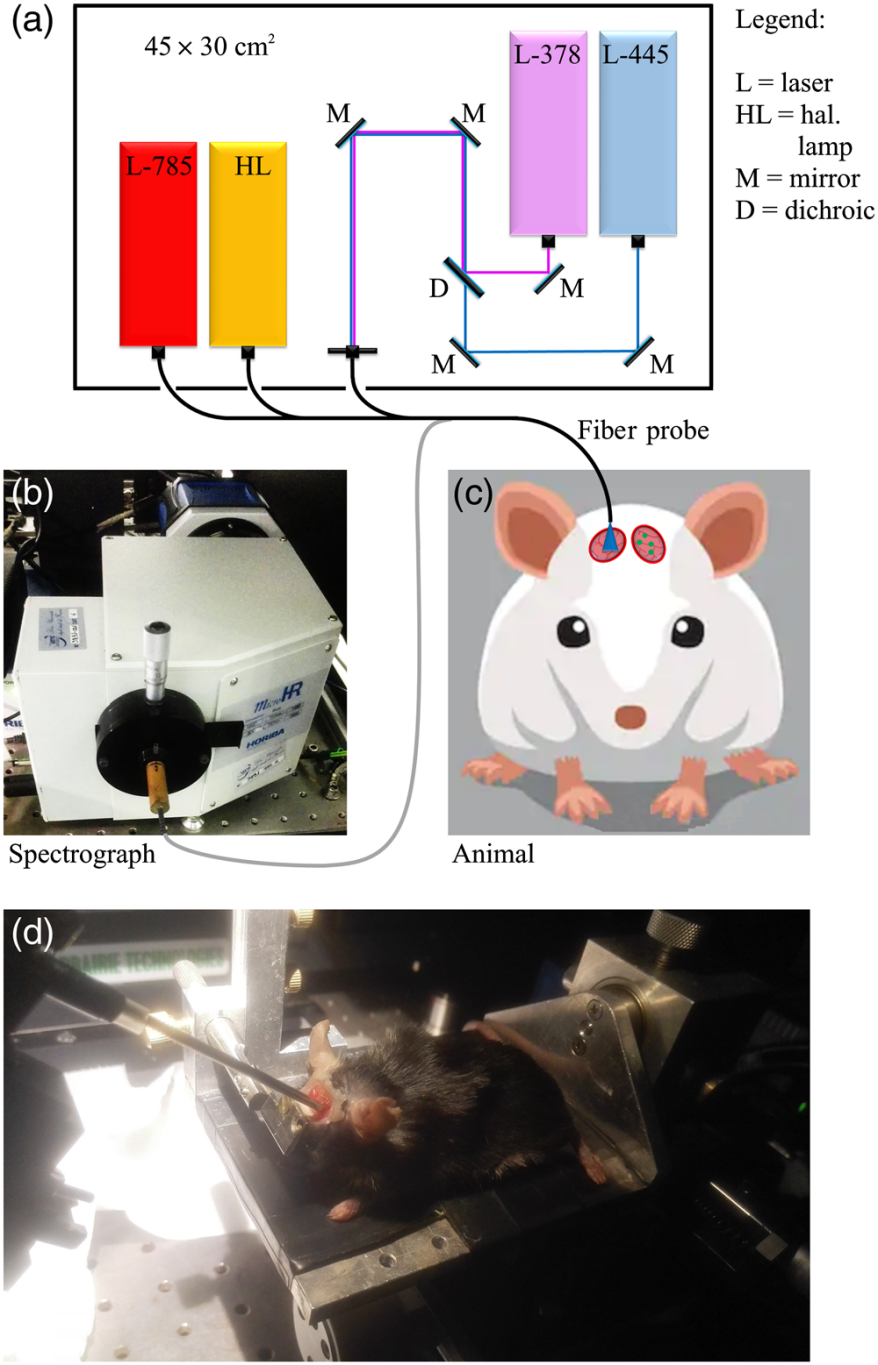
(a)–(c) Simplified scheme of the experiment. (a) A quadri-furcated fiber probe is connected to four excitation sources contained in a $45\times 30\;{\mathrm{cm}}^{2}$ box. (c) The excitation light is delivered through an optical window on the mice skull. (b) The generated signal (Raman/flurescence/reflectance spectra) is backward collected and sent to the spectrograph. (d) Picture of an anesthetized laboratory mouse during the experiment with the probe tip pointing toward the skull window.

Eight animals were included in this study. After two weeks from the injection of EGFP-GL261 cells, each mouse was anesthetized and placed under the stereotaxic device (Fig. 1) for examination through our optical fiber probe (Sec. 2.3.1), whose distal end was placed perpendicularly in contact with each of the two quartz windows. Few animals were inspected also in the following days, in order to record tumor progression; in total, we performed 13 experiments.

First, fluorescence spectroscopy was used for real-time (<0.1  s) detection of tumor areas by taking advantage of the characteristic EGFP signal. The probe was placed in the point of maximum EGFP intensity; then, fluorescence, Raman, and reflectance spectra were acquired from the same region. Spectral measurements were performed also on the second optical window (i.e., on control brain tissue). Total integration times were selected in order to exploit the dynamic range of the CCD and to reduce noise. For fluorescence acquisitions, they ranged between 0.5 and 5 s—depending on the presence of EGFP and the amount of autofluorescence—obtained by averaging 10 recordings and using 5- and 3-mW excitation power at 378 and 445 nm, respectively. Each Raman measurement required averaging 10 recordings, resulting in 25- to 50-s integration times using 75-mW power. Reflectance spectra were acquired by averaging 100 recordings in 0.1 to 5 s, using ∼5-mW typical output power (from a nominal bulb power of ∼5  W). The inspection typically required a total of less than 4 min for each animal, resulting in 1 spectrum for each spectroscopic technique and for each region. Tumor areas were later examined through a multiphoton microscope (Sec. 2.3.2) in order to observe EGFP-labelled cells and to estimate tumor size.

### 2.2 Pellet Preparation and Measurements

We investigated the presence of EGFP spectral contributions in Raman and reflectance measurements by examining two pellets with our fiber probe: one made of wild-type (WT) GL261 cells and the other one of EGFP-GL261 cells.

Each pellet was produced starting from two 100-mm tissue culture dishes containing GL261 cells that reached 100% confluence. The medium was discarded from the dishes and then they were first washed with Dulbecco phosphate-buffered saline (DPBS) without calcium and magnesium (Gibco 14190136). Trypsin-EDTA 0.05% (Gibco 25300054) was added to the dishes, and then cells were incubated for 2 to 3 min at 37 °C. After that, trypsin was blocked by adding cell culture medium and cells were resuspended to a single-cell suspension by pipetting with a 1000-ml pipette. Then the cells were transferred to a 15-ml conical tube and centrifuged at 200 g. Then the pellet was measured in a 4% paraformaldehyde solution.

### 2.3 Experimental Setups

#### 2.3.1 Fiber-probe system

Spectral measurements were performed through the experimental setup described in Ref. 6. As shown in Fig. 1, this system consists of four excitation sources, a quadri-furcated fiber probe (EMVision LCC, Loxahatchee, Florida), and a spectrograph (MicroHR, HORIBA Scientific, Edison, New Jersey) equipped with a 600-lines/mm grating and with an open-electrode CCD camera (Syncerity, HORIBA Scientific, Edison, New Jersey) cooled at −60°C.

In particular, we used two laser diodes (TEC 042, Sacher Lasertechnik, Maburg, Germany) for exciting fluorescence at 378 and 445 nm, respectively; a narrow-band laser diode at 785 nm (FC-785-350-MM2-PC-1-0-RM, RGBLase, Fremont, California) for near-infrared (NIR) Raman spectroscopy; and a halogen lamp (HL-2000-LL, Ocean Optics, Dunedin, Florida) for reflectance spectroscopy.

The probe, which is connected to all light sources, consists of a bundle of 11 optical fibers: a central fiber (300-μm core diameter) for delivering 785 nm; 7 surrounding fibers (300  μm each) for Raman collection; 2 fibers (200  μm each) for delivering 378/445 nm and white-light from the lamp, respectively, and 1 fiber (200  μm) for collecting both fluorescence/reflectance signals, all of them placed near to the central fiber. A narrow bandpass filter centered at 785 nm was placed in front of the Raman delivering fiber in order to clean the laser line, blocking unwanted fluorescence and Raman signals generated inside the optical fiber itself. A ring-shaped long-pass optical filter was placed at the distal end of the probe, preventing 785-nm laser light from entering the seven collection fibers. A motorized filter wheel allows changing emission filter according to the spectral technique used: a long pass filter at 400 nm (Longpass 400 nm, Melles Griot, Albuquerque, New Mexico) for fluorescence spectroscopy excited at 378 nm; a long pass at 458 nm (LP02-458RS-25, Semrock, Rochester, New York) for fluorescence spectroscopy excited at 445 nm; a long pass at 785 nm (LP02-785RE-25, Semrock) for Raman spectroscopy; and a neutral density filter with OD=0.3 for diffuse reflectance. These filters prevent laser light from entering the monochromator and lamp light from saturating the detector.

The device was designed to be easily handled (as a pen) for fast examination of suspicious tissue areas.

#### 2.3.2 Two-photon excited fluorescence microscope

High-resolution two-photon excited fluorescence (TPEF) *in vivo* imaging was performed on a Prairie Ultima Multiphoton microscope equipped with a mode-locked Ti:sapphire laser (Chameleon Ultra II, Coherent, Santa Clara, California). Laser excitation was set at 910 nm for EGFP detection and a maximum power of ∼35  mW was used for data acquisition. Images were acquired with a water immersion lens (XLUMPLFLN20XW, Olympus, Tokyo, Japan; 20× magnification, NA=1) at 1024×1024  pixel resolution and zoom = 1, leading to 600  μm field of view with linear resolution of 0.594  μm/pixel.

### 2.4 Data Analysis

#### 2.4.1 Pre-processing of spectra

About 26 spectra—13 from healthy mice brains and 13 from GBM tissues—were recorded for each optical technique (fluorescence excited at 378 and 445 nm, Raman, reflectance). The transmission spectrum of the fiber-probe setup was used for correcting all the recorded spectra in order to remove artifacts. Also, further processing was performed on Raman and reflectance datasets. In particular, we used a sixth-order polynomial fitting through an automated routine (Vancouver Raman algorithm^17^) for removing NIR fluorescence and background signals from the Raman spectra, which were later normalized to the ${1445\text{-}\mathrm{cm}}^{-1}$ Raman band (corresponding to ${\mathrm{CH}}_{2}$ and ${\mathrm{CH}}_{3}$ deformations^18^) intensity. Reflectance spectra were corrected for the lamp emission spectrum and normalized to the intensity recorded at 570 nm, which is one of the oxy/deoxy-hemoglobin isosbestic points.

#### 2.4.2 Spectral classification analysis

This study aims to discriminate healthy brain and glioma tissues in a label-free modality, e.g., based on the information recorded through Raman and reflectance spectroscopies. In this framework, PCA and LDA are well-known methodologies^19^^–^^21^ used for the classification of spectral data;^6^^,^^22^^,^^23^ we implemented each method in a separate custom-made routine on LabVIEW (National Instruments, Austin, Texas). PCA was used for reducing noise and redundant information by selecting four principal components (PCs) of each dataset and discarding all the others. Also, a decreased dimensionality reduces the risk of overfitting the recorded data and, thus, overestimating classification accuracy. LDA, then, was employed to build a linear combination of the selected PCs in order to maximize discrimination between different tissue classes. We specifically designed an LDA routine for generating a classifier and maximizing the area under its receiver operating characteristic curve (auROCc), a parameter useful for estimating the classification accuracy.^24^^,^^25^ For each spectrum, the algorithm creates a linear combination by multiplying each selected PC loading by a random number between 0 and 100, then summing all the four products into a score. The ROC curve of normal and tumor scores and the corresponding auROCc values are then calculated and recorded. The algorithm iterates such routine, each time comparing the new auROCc value with the maximum value among those ones already calculated, and stops after N consecutive iterations (e.g., N=500) where no further maximum is found. Finally, the linear combination providing the best accuracy is saved and can be used to classify a group of unknown spectra. A cross-validation function can be selected for dividing the dataset in (k)-folds, using (k−1)-folds for training the classification algorithm and 1 fold for testing it, and repeating the process (k)-times by rotating the folds in the two groups.

Two additional analytical methods were applied to the recorded spectra: ratiometric scoring (i.e., calculating the ratio between photon counts recorded at two different wavelengths/wavenumbers) and slope analysis (i.e., applying a linear fit to a region of the spectrum in order to derive its slope). Both analyses were implemented in LabVIEW.

#### 2.4.3 Image analysis

TPEF images were reconstructed and analyzed through ImageJ (National Institutes of Health, Bethesda, Maryland). The radial (i.e., parallel to brain surface) extension of each tumor was estimated by measuring the average distance between opposite margins. Instead, tumor axial (i.e., perpendicular to brain surface) extension could not be estimated due to the limits in NIR light penetration (and visible fluorescence back-propagation) in highly scattering media such as brain tissue; roughly, the microscope was not able to image deeper than 400  μm.

## 3 Results

### 3.1 EGFP Fluorescence, Raman, and Reflectance Spectra

**Fig. 2:**
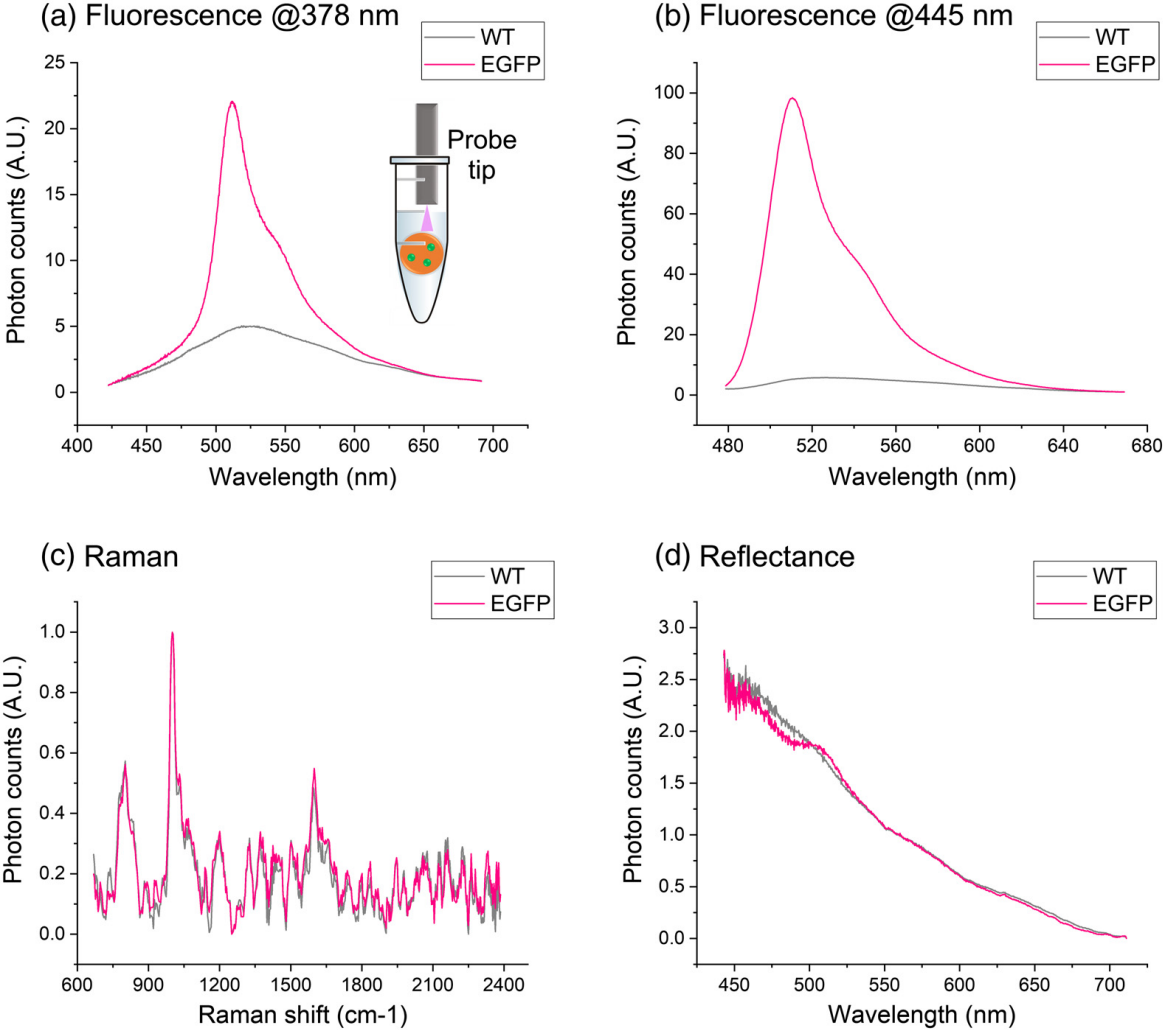
Spectral results on WT (gray) and EGFP (pink) cell pellets: fluorescence spectra excited at (a) 378 nm and (b) 445 nm (b), (c) Raman, and (d) reflectance spectra.

We used the fiber-probe system for spectroscopic examination of both WT- and EGFP-GL261 pellets. Figure 2 shows our results. As expected, UV- and blue-excited fluorescence spectra of the EGFP pellet [Figs. 2(a) and 2(b), respectively] are dominated by the label emission peak at 510 nm; instead, autofluorescence signal is relatively negligible. Moreover, we found stronger EGFP signal in blue-excited spectra, as their excitation wavelength (445 nm) is closer to the maximum absorption peak (490 nm) of the protein.

**Fig. 3:**
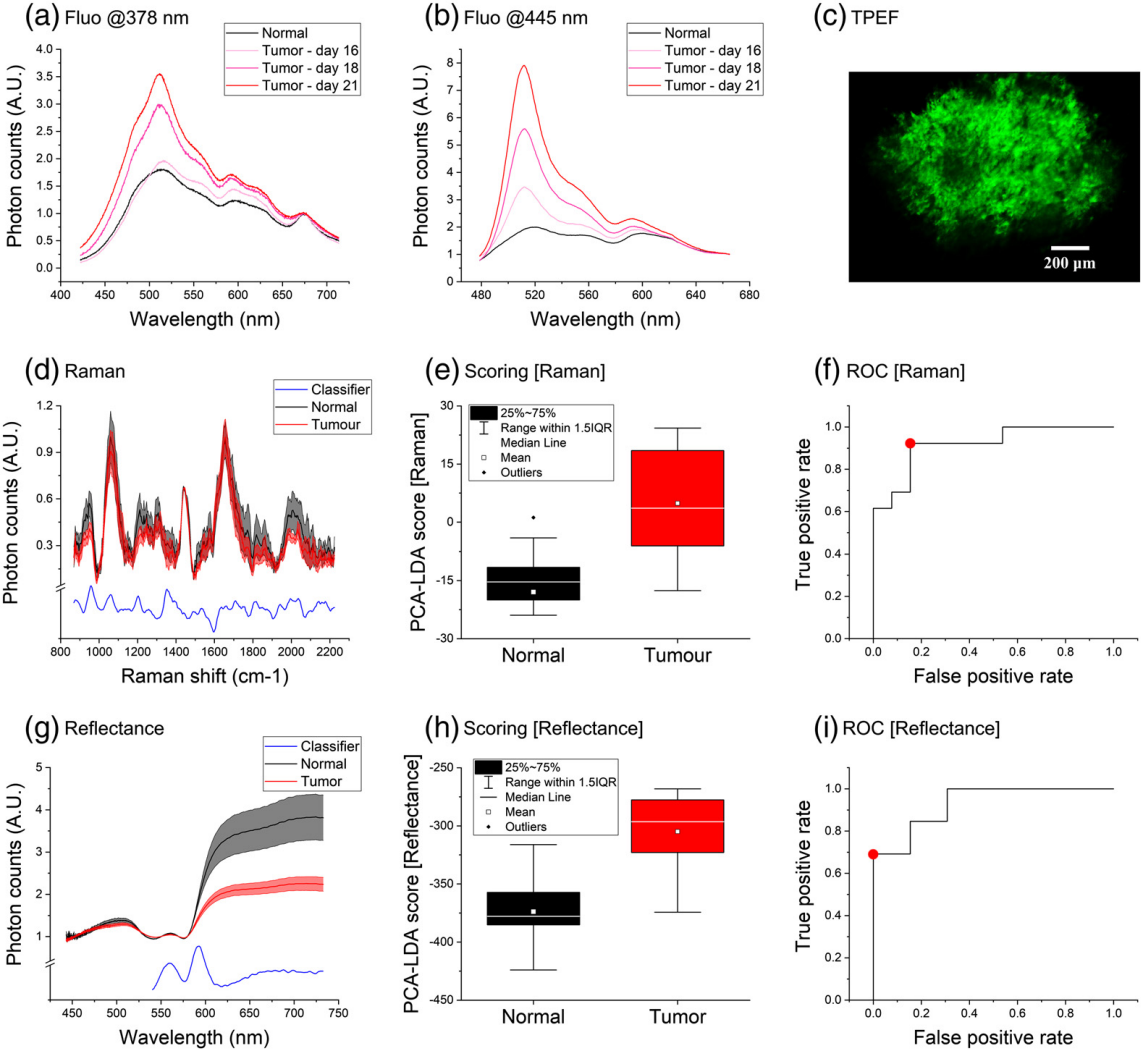
(a) and (b) Time lapse of fluorescence spectra—excited at 378 and 445 nm, respectively—collected from healthy (black) and tumor (light pink, dark pink, and red) brain tissues within the same laboratory mouse. (c) TPEF mapping of murine GBM labelled with EGFP. Mean (d) Raman and (g) reflectance spectra ± standard errors of normal (black) and tumor (red) murine brain tissues, averaged over 13 observations on 8 mice, compared to the two corresponding classifiers (blue) based on PCA-LDA analysis. Results of the scoring algorithm applied to each spectral dataset, presented as normal/tumor (black/red) scores generated by the (e) Raman and (h) reflectance classifiers. ROC curves of the (f) Raman and (i) reflectance classifiers; each red dot corresponds to a suggested threshold for discriminanting normal and tumor scores.

Raman spectra [Fig. 2(c)] are mainly characterized by the phenylalanine bands^26^^,^^27^ at 1000, 1030, and $1600\;{\mathrm{cm}}^{-1}$, showing no significant differences between WT and EGFP cell pellets. Instead, reflectance spectra have different trends in the 450 to 500 nm and 500 to 540 nm ranges—corresponding, respectively, to EGFP absorption and emission—while the two signals show good correspondence at longer wavelengths and mostly overlap. Hence, we found that EGFP presence does not affect Raman measurements through our setup, but it may do so in reflectance recordings. Although the effects of the fluorescent label are not clearly visible in tissue reflectance acquisitions [Fig. 3(g)], we decided to restrict the analysis of those spectra (Sec. 3.2) in the 540 to 733 nm range.

### 3.2 Brain Tumor Detection in Mice

#### 3.2.1 Tumor localization

Tumor areas in mice brain were detected through their EGFP emission peak excited at 378 and 445 nm [Figs. 3(a) and 3(b), respectively]. In fact, after placing each animal on the stereotaxic stage, we continuously acquired fluorescence spectra while moving the fiber probe along the tumor window; then, we positioned the probe where the fluorescent label signal was maximized. We found that EGFP increased day by day, as tumor size increased over time; also, we recorded the autofluorescence spectrum of healthy brain tissues from the center of the other quartz window for a qualitative comparison against spectra of EGFP-labelled tumor. However, only a small number (maximum 3) of time points was recorded for each animal, due to the limited time window—few days—available for measurements before its death; moreover, in order to reduce the stress for the animal, a 1-day pause was interposed between every two sessions.

**Fig. 4:**
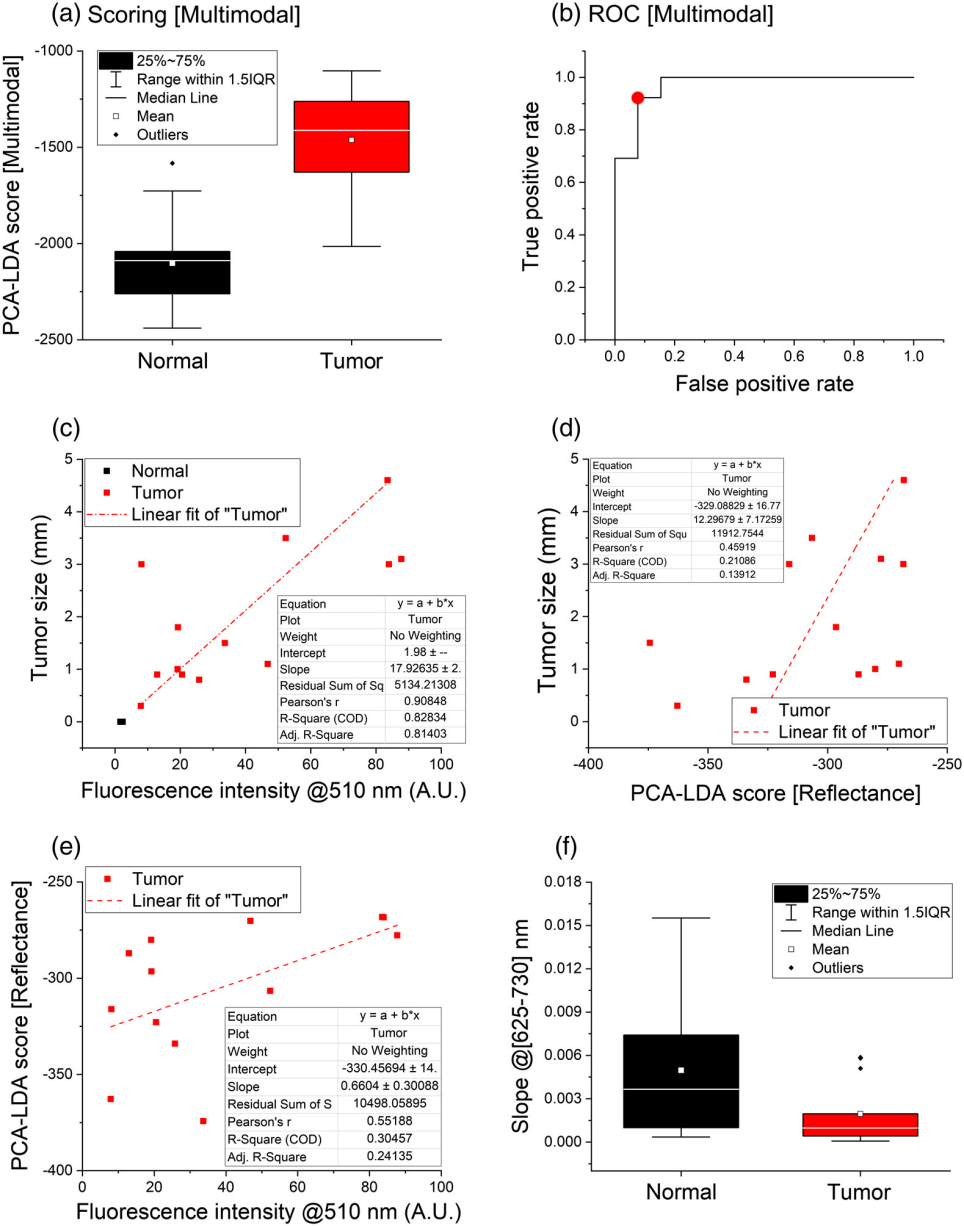
(a) Results of the scoring algorithm based on the linear combination of Raman and reflectance PCA-LDA scores, presented as normal/tumor (black/red) scores generated by the classifier. (b) ROC curves of the multimodal classifier; the red dot corresponds to a suggested threshold for discriminanting normal and tumor scores. (c) and (d) Correlation of the tumor radial size with, respectively, the EGFP peak intensity and with the scores of the PCA-LDA classifier applied to reflectance data. (e) Correlation of the EGFP peak intensity with the reflectance scoring. A linear fit (dash-dotted red line) of the tumor datapoints was superimposed for each dataset presented; in graph (c), the intercept was fixed to the mean value of normal datapoints. (f) Slope values of normal (black) and tumor (red) refletance spectra calculated in the 625 to 730 nm range.

Tumor radial extension was correlated with fluorescence intensity excited at 445 nm and emitted at 510 nm, corresponding to the EGFP emission peak [Fig. 4(c)]. These variables seem to be related ($R^{2}=0.828$, Pearson’s r=0.908 for a linear fit): normal dots are clustered in one small area, while tumor dots follow an approximately linear trend. However, there are few considerations to be made. In the first place, peak intensity is calculated relatively to tissue autofluorescence emitted at 660 nm; thus, biological variation may introduce a certain degree of “noise” in the reported values. Second, the intensity of EGFP emission depends on the spatial convolution between tumor volume (i.e., its radial and axial extensions) and the excitation volume of the probe. Since the radial laser spot is $∼1\;{\mathrm{mm}}^{2}$, fluorescence intensity for tumors larger than that size depends only on their axial extension; it can be expected that, in such cases, EGFP intensity increases with tumor depth. Then, if radial and axial extensions grow proportionally together, this is a possible explanation for seeing a linear trend in Fig. 4(c).

#### 3.2.2 Raman results

We recorded two Raman spectra from each animal inspection, i.e., from both healthy and tumor brain regions. Figure 3(d) shows the average spectra of these tissue types, which are characterized by the typical Raman peaks of amino acids (such as proline and valine bands^18^ at $950\;{\mathrm{cm}}^{-1}$), nucleic acids^28^ (1060 and $1575\;{\mathrm{cm}}^{-1}$), lipids ($1445\;{\mathrm{cm}}^{-1}$), amide III and tryptophan^29^ ($1210\;{\mathrm{cm}}^{-1}$), and amide I^27^ ($1650\;{\mathrm{cm}}^{-1}$). By normalizing spectral intensity to the ${1445\text{-}\mathrm{cm}}^{-1}$ band, the major differences seem to reflect higher amino acids, tryptophan, and amide III relative (i.e., with respect to the lipid content) concentrations in healthy brain, and higher amide I relative concentration in tumor tissues. These findings could be partially explained with the altered cell metabolism in gliomas, which causes an increased rate of lipid synthesis during their growth^30^ and lower levels of amino acids.^31^ A classifier based on ratiometric scoring resulted in 77% and 79% auROCc (data not shown) by calculating the ratio between Raman photon counts registered at the ${1450\text{-}\mathrm{cm}}^{-1}$ and, respectively, 985- and ${2030\text{-}\mathrm{cm}}^{-1}$ bands.

The first 15 PCs of the Raman dataset were calculated through standard PCA. We selected four of them, based both on the discrimination capability of each one (evaluated through its auROCc) and the improvement provided to the PCA-LDA analysis (again, measured as increased/decreased auROCc value). A classification model was generated from the linear combination of PC3, PC7, PC10, and PC13; the resulting classifier was denoised and reported in Fig. 3(d) in order to show the correlation between its major peaks and the observable differences between normal and tumor spectra. Finally, each Raman spectrum was assigned a “score” [Fig. 3(e)] based on the classifier, whose auROCc was found to be 92% [Fig. 3(f)] when training the algorithm on all dataset spectra. Then, in order to validate this result, we performed an automated 4-fold cross-validation (4f CV) analysis—10 normal and 10 tumor samples for training, 3 normal and 3 tumor samples for testing—resulting in 86% average auROCc (see Table 1).

**Table 1:** Specificity/sensitivity and auROCc values obtained using the classification models based on PCA-LDA. First and second rows show the results from the separate classification of Raman and reflectance datasets, respectively, while the third row reports their combined analysis. The last column on the right reports auROCc values obtained through 4f CV.

	Specificity/sensitivity (%)	auROCc (%)	4f CV auROCc (%)
Raman	85/92	92	86
Reflectance	100/69	93	92
Multimodal	92/92	97	95

#### 3.2.3 Reflectance results

Figure 3(g) shows the average reflectance spectra recorded from normal murine brain and GL261 tumor areas. Each spectrum was normalized to an isosbestic point of oxy- and deoxy-hemoglobin (570 nm) in order to take advantage of their different molar extinction coefficients, to qualitatively compare their relative abundance in the two examined tissue types and, hence, to derive information on the corresponding oxygen levels. In fact, one of the most characteristic features of these spectra regards oxy-hemoglobin absorption peaks^32^ (540 and 575 nm), indicating that such substance is one of the major absorbers. Normal and tumor spectra intersect each other also in the others’ isosbestic points^33^ (452, 529, 545, and 584 nm), with the only exception of 500 nm. It is unclear whether the presence of EGFP may be partially responsible for missing the latter intersection, because a similar behavior can be observed in Ref. 14 between label-free normal and cancer brain tissue spectra.

The major differences in Fig. 3(g) take place between 600 and 730 nm: murine gliomas show higher relative absorption in this region, suggesting a higher concentration of deoxy-hemoglobin with respect to oxy-hemoglobin, thus lower oxygenation with respect to normal brain. Indeed, this is consistent with the typical hypoxic environment^34^ of tumors. These patterns are so prominent that a classifier based on ratiometric scoring resulted in 82% auROCc (data not shown) by calculating the intensity ratio between 570 and 690 nm.

Another feature can be observed at longer wavelengths (>625  nm), where healthy spectra have slightly higher slope than tumors. It is known^35^ that cell nuclei dimension affects reflectance spectra in this region, specifically with respect to the reduced scattering coefficient (which is inversely proportional^36^ to the square root of scatterers diameter). Thus, as reflectance intensity is an approximately linear^37^ function of such coefficient, the ratio between normal and tumor spectral slopes could reflect their different nuclear size. We implemented a linear fitting algorithm in LabVIEW for deriving normal and tumor slopes between 625 and 730 nm [Fig. 4(f)], finding a normal-to-tumor slope ratio of 2.6±1.5. Formally, this value is consistent with typical tumor-to-normal root square nuclear size ratio (for example, we calculated a 1.25±0.04 ratio from data reported in Ref. 38), although the reported standard error is relatively high. A classifier based on slope values resulted in 73% auROCc.

Finally, the same PCA-LDA methodology used for the analysis Raman spectra was applied to the reflectance dataset. The first 15 PCs were calculated through standard PCA, then 4 of them (PC1 to PC3 and PC5) were used to generate a classification model. The PCA-LDA classifier [reported, after denoising, in Fig. 3(g)] reached 93% auROCc [Table 1, Figs. 3(h) and 3(i)] when training the algorithm on all dataset spectra; a 4f CV resulted in almost the same accuracy (92%). Moreover, we evaluated the correlation between our model and tumor diameter [Fig. 4(d)] or EGFP intensity [Fig. 4(e)]. Using a linear fit, a positive correlation was observed in both cases (Pearson’s r=0.459 and 0.552, respectively); however, the regression line did not result in good fitting of the data ($R^{2}=0.211$ and 0.305). Thus, the classifier based on reflectance spectroscopy seems capable of detecting the tumor and potentially sensitive to its diameter, although more experiments and training of the model would be required to quantitatively evaluate its size.

#### 3.2.4 Multimodal results

We reached high sensitivity (92%) in classifying Raman spectra and high specificity (100%) for reflectance ones. Since Raman and reflectance spectroscopies provide complementary information, combining their results could improve tissue characterization. Thus, the scores resulting from their classification models were linearly combined through the same LDA routine in order to maximize discrimination between normal and tumor areas. We found a sensible increase in auROCc value (97% from the whole dataset and 95% from the cross-validation), while the suggested threshold corresponded to 92% sensitivity and specificity.

Tumor multimodal scores were also correlated to GBM size and EGFP signal, but the results from both linear fits were worse than the corresponding analyses on reflectance spectra. Such outcome reflected the fact that Raman scores were totally uncorrelated to tumor dimensions, hence their contribution to multimodal scoring reduced the correlation observed from reflectance data.

## 4 Discussion

Surgical resection plays a major role in current GBM treatment, hence the necessity of improving the detection of brain tumor areas in a fast and reliable way is key to a positive prognosis. Multimodal optical spectroscopy could be a practical tool for such purpose, as demonstrated in this preliminary study, where we employed *in vivo* Raman and reflectance spectroscopies in order to discriminate healthy brain from tumor tissue in a mouse GBM model. This approach led to the classification of healthy and tumor tissues in a label-free modality, based only on their intrinsic molecular composition (Raman) and oxygenation (reflectance). Our findings suggest that these spectroscopic techniques are able to recognize the altered metabolism of mouse glioma and its hypoxic environment.

In particular, Raman spectroscopy could be used to automatically evaluate lipids and amino acids concentrations from the analysis of the fingerprint region, as they seem to be important biomarkers for brain tumor recognition. It should be noted that these results are consistent with 10 years of successful Raman applications^7^^,^^39^^–^^42^ (both *ex vivo*^43^ and *in vivo*^14^) to the detection of brain tumors, of whom many studies involving handheld fiber probes such as ours and involving similar numbers of patients/subjects. For example, Jermyn et al.^39^ and Jermyn et al.^14^ used fiber-probe Raman spectroscopy for *in vivo* detection of human brain tumors from 17 and 15 patients, respectively, obtaining ≥95% auROCc. More generally, Zhang et al.^42^ summarized the results from 6 studies—mostly *ex vivo*—performed between 2005 and 2015, concluding that Raman spectroscopy provided 98% auROCc for glioma discrimination. While there are similarities between the presented study and previous works, there are also notable differences regarding number of acquisitions per subject (e.g., 10 or more spectra, instead of a single one) and detection instrumentation (i.e., CCDs with higher sensitivity and cooled down to lower temperatures), which may explain the comparatively lower signal-to-noise ratio of our spectra. Another major difference regards the analytical methods adopted, which can strongly affect classification accuracies; for instance, we found the analysis of Raman spectra to be sensibly dependent on the criteria for PCs selection. Finally, the presented experiment was designed differently from the known literature, as EGFP-labelling was used in order to precisely localize murine GBM areas and to record their Raman signatures. In our view, this approach could be followed in other oncologic studies based on animal models.

Reflectance spectroscopy has been used for glioma detection, too, either in single modality^44^^,^^45^ or multimodal combination^11^^,^^13^^,^^14^ with fluorescence and/or Raman. Some studies, for example, used optical parameters derived from reflectance spectra (e.g., attenuation, absorption, and scattering coefficients) in order to correct the corresponding normal and tumor fluorescence spectra. Jermyn et al.^14^ improved *in vivo* brain cancer detection up to 98% auROCc by analyzing both Raman and attenuation-corrected autofluorescence spectra. Similarly, Valdes et al.^11^ applied absorption/scattering corrections to fluorescence spectra collected *in vivo* from 10 patients; notably, a fluorescence biomarker (PpIX) was used in order to provide contrast between control and tumor areas, resulting in 94% auROCc from the analysis of corrected fluorescence recordings. Other works, instead, discriminated brain tumors by directly analyzing reflectance data. Lin et al.^45^ analyzed reflectance spectra collected from 12 patients, obtaining 77% auROCc in control/tumor discrimination by looking at the normalized intensity recorded at 820 nm. Du Le et al.^13^ examined *ex vivo* samples excised from 7 patients, obtaining 100% sensitivity and 80% specificity in discriminating GBM from low-grade gliomas by looking at the intensity recorded at 650 nm. Therefore, the results presented in this study are consistent with the known literature. In fact, we successfully applied reflectance spectroscopy for probing tissue oxygenation and, also, deriving information on scatterers’ size. Moreover, the stark and label-free contrast between normal and diseased reflectance spectra allowed developing a classification algorithm with high accuracy. Further investigation of brain tissues could involve measuring absolute absorption, since tumors typically stimulate the formation of new blood vessels (angiogenesis), but the methodology applied in this work was not enough standardized to perform such a task.

The use of a halogen lamp as excitation source represents a simpler and cheaper solution with respect to laser-based techniques; moreover, both detection system and data analysis could be eventually simplified into a narrow-band ratiometric approach. Considering the high speed of these spectral recordings and its relatively low implementation cost, such technique offers an enormous translational potential as it fits with all the requirements for a clinical deployment. However, the known literature and this study suggest that a multimodal approach—e.g., combining reflectance with laser spectroscopy—is crucial for improving sensitivity and specificity in tumor detection beyond the capability of each single technique. Further, multimodal spectroscopy could be exploited for scanning large brain areas upon implementation within real-time fiber probes^46^ or imaging systems.^47^

In conclusion, the *in vivo* results presented here represents an additional, although preliminary, encouraging step toward clinical translation and deployment of Raman and reflectance spectroscopies for a fast, label-free detection, and delineation of human brain tumors.
